# Comparison of thyroid fine needle aspiration biopsy and ultrasonography results

**DOI:** 10.1097/MD.0000000000033822

**Published:** 2023-06-30

**Authors:** Lezzan Keskin, Doğu Karahan, Bülent Yaprak

**Affiliations:** a Turgut Ozal University, Malatya Training and Research Hospital, Internal Medicine Department, Malatya, Turkey.

**Keywords:** bethesda, biopsy, nodule, thyroid

## Abstract

Thyroid nodules are one of the most common health problems in the community. Although most of the nodules are benign, Fine needle aspiration biopsy (FNAB) is requested due to malignancy concerns. In this research, the aim was to make a comparison of the results of thyroid ultrasonography (USG) and FNAB for thyroid nodules. This study was conducted retrospectively on the data of 532 patients. Detail Edu ultrasonographic assessment was conducted before the FNAB procedure and FNAB was performed by an endocrinology specialist. FNAB results and Thyroid USG features were compared, and thyroid FNAB results were graded using the classification of World Health Organization Bethesda-2017. The average age of the individuals included in the research was 49.99 ± 13.65 (min = 18-max = 97). According to the 2017 Bethesda classification, 74.6% of FNAB results were benign, 16% follicular lesion of undated mined significance or A type of undated mined significance, 0.9% were malignant, and 1.1% were suspicious for malignancy. When USG findings were compared according to FNAB results, it was found that malignant lesions were significantly higher in single nodules (non- cystic and non- mixed lesions). Lesions with a single nodule on USG were found to be 3.6 times more likely to be malignant (OR 95% CI: 1.172–11.352). The gold standard method for the diagnosis the presence of thyroid nodules is thyroid fine needle aspiration biopsy with ultrasound guidance. Taking samples from the correct nodule and component increases its value. The presence of a single nodule from the thyroid USG features was found to be an important predictor of malignancy according to the biopsy results.

## 1. Introduction

Nodules on the thyroid are a typical medical issue. In places of the world where iodine is abundant, epidemiological studies have revealed that the prevalence of palpable thyroid nodules is roughly 1% for males and 5% for females.^[1,2]^ On the contrary, thyroid nodules can be found by high resolution ultrasonography (USG) in 19% to 68% of randomly chosen people. Thyroid nodules tend to affect the seniors and females more frequently.^[3]^

The necessity to exclude out thyroid cancer, which develops in 7% to 15% of cases according to age, family history, gender, history of exposure to radiation, and other factors, underlies the clinical significance of thyroid nodules.^[4,5]^ Although most of the thyroid nodules are benign, detection of nodules on USG causes concern in patients and increases admissions to the endocrinology department. Fine needle aspiration biopsy (FNAB) in thyroid nodules is an easy method to evaluate together with USG findings, and it makes a positive contribution to the decision of surgery or follow-up with the 2017 Bethesda Thyroid Cytopathology Reporting System criteria in the malignant and benign differentiation.^[6]^

Our study aimed to compare the results of thyroid USG and FNAB in individuals having thyroid nodules detected in the endocrinology outpatient clinic. In this way, it was planned to evaluate the importance of thyroid USG and FNAB, which is the gold standard method in the differentiation of benign and malignant thyroid nodules.

## 2. Material and method

This study was carried out on patients who applied to Malatya Turgut Özal University Faculty of Medicine Education and Research Hospital Endocrinology and Internal Medicine outpatient clinic. The study was designed retrospectively. Patient files between December 2021 and August 2022 were scanned, and data of 532 patients were obtained as a result of the scan. The Local Ethics Committee of the Turgut Ozal University granted permission for the study to be carried out (Decision no: 2022/49). Individuals with thyroid stimulating hormone levels < .1 mIU/L and who underwent surgery for toxic nodules were not included in the investigation. The study excluded individuals who had neck and head radiation and females who were pregnant. Patients with euthyroid nodules with no radioactive iodine uptake on scintigraphy, and nodules with a nodule size > 10 mm or < 10 mm with additional risk factors (family history, microcalcification, irregular border) were included in the research.

### 2.1. Thyroid ultrasonography and fine needle aspiration biopsy

Detailed ultrasonographic evaluations of the patients were conducted prior to the FNAB procedure. Afterwards, FNAB was conducted by an endocrinologist. Thyroid USG was performed by expert radiologists utilizing 5 to 12 MHz linear array transducers (GE LOGIQ 7). Ultrasonographic features; echo structure (mixed and solid), nodule size, echogenicity (mixed echogenicity, hyperechogenicity or isoechogenicity, hypoechogenicity), nodule structure (solid, mixed solid-cystic, spongioform, cystic), edge (irregular or regular), halo (absent, limited) or unrestricted and the absence/presence of microcalcifications. A nodule with <50% of its total surface area degenerated into cysts was referred to as a mixed solid-cystic nodule. A nodule with mixed echogenicity is one that has both isoechoic and hypoechoic regions.

### 2.2. Cytological evaluation

All histopathological examinations were performed by a pathologist. Two thousand seventeen Bethesda Thyroid Cytology results are classified according to the Cytopathology Reporting System:

(i) nondiagnostic or inadequate.
(ii) Benign.
(iii) Atypia of uncertain significance O or follicularUlesion of uncertain significance.
(iv) Follicularaneoplasm or a follicular suspected foraneoplasm.
(v) Suspected forlmalignancy.
(vi) Malignant.

All thyroid tissue samples were embedded in paraffin and stained with hematoxylin and eosin after being fixed in 10% neutral buffered formalin. Results from the FNAB and USG were contrasted. Based on the 2017 World Health Organization classification, thyroid carcinoma was categorized.

### 2.3. Statistical analysis

The data analysis was done using the SPSS-22 program. Shapiro Wilks test was used as the normal distribution test. The t test was utilized in the analysis of normally distributed data, and the Chi-Square test was employed in the comparison of categorical data. Regression analysis Binary logistic was employed for further analysis. A *P* value of < .05 was accepted statistically significant.

## 3. Results

The average age of the individuals included in the research was 49.99 ± 13.65 (min = 18-max = 97) and 455 (85.5%) of them were male, 77 (14.5) of them. When USG findings were compared according to FNAB results, it was found that malignant lesions were statistically significantly higher in single nodules, non-cystic solid nodules and non-mixed lesions. The comparison of the USG characteristics of the patients according to the demographic characteristics and biopsy results is given in Table 1 (The nondiagnostic group was excluded from the analysis in Table 1 analysis).

**Table 1:** Comparison of features according to FNAB result.

	FNAB Result n(column%) or X ± SD		*P* value
	Benign	Malignant	
Age	50.01 ± 13.65	47.85 ± 14.29	.573
Gender (Y/F)	73 (15.1)/409 (84.9)	1 (7.7)/12 (92.3)	.702
BMI	29.22 ± 5.37	31.95 ± 5.80	.322
Nodule features			
Echogenicity			
Isoechoic	134 (27.8)	5 (38.5)	.410
Hypoechoic	136 (28.2)	4 (30.8)	
Hyperechoic	22 (4.6)	1 (7.7)	
Heterogeneous	190 (39.4)	3 (23.1)	
Nodule number			
Only	104 (21.6)	7 (53.8)	.012
Multiple	378 (78.4)	6 (46.2)	
Solid			
No	11 (2.3)	0 (0)	1000
Yes	471 (97.7)	13 (100.0)	
Cystic structure			
No	237 (49.2)	11 (84.6)	.021
Yes	245 (50.8)	2 (15.4)	
Calcification			
No	373 (77.4)	10 (76.9)	.578
Microcalcification	91 (18.9)	2 (15.4)	
Macrocalcification	18 (3.7)	1 (7.7)	
Wall structure			
Faint	47 (9.8)	1 (7.7)	.888
Irregular	68 (14.1)	1 (7.7)	
Regular	367 (76.1)	11 (84.6)	
Smoking history			
No	425 (88.4)	12 (92.3)	1000
Yes	56 (11.6)	1 (7.7)	
Family history of thyroid cancer			
No	470 (97.5)	13 (100.0)	1000
Yes	12 (2.5)	0 (0)	
Mix			
No	249 (51.7)	11 (84.6)	.023
Yes	233 (48.3)	2 (15.4)	

FNAB = fine needle aspiration biopsy.

In our study, according to the 2017 Bethesda classification, 74.6% of the patients FNAB results were found to be benign, 16% to have atypia of uncertain significance, 0.9% to be malignant, and 1.1% to suspected malignant (Table 2).

**Table 2:** FNAB results according to 2017 Bethesda classification.

Diagnosis	n	%
Non-diagnostic	37	7.0
Bening	397	74.6
Atypia of undetermined significance (AUS) or follicular lesion of undated mined significance (FLUS)	85	16.0
Follicular neoplasm (or suspicious for follicular neoplasm)	2	0.4
Suspicious for malignancy	6	1.1
Malignant	5	0.9
Total	532	100.0

FNAB = fine needle aspiration biopsy.

It was found that the logistic regression analysis, which was created to predict the results of FNAB by utilizing the ultrasonographic features of the nodules, is important (Omnibus test *P* = .008). The independent variables of the model are the number of nodules (ref: multiple, risk: single), the cystic structure status (ref: cystic, risk: not cystic) and the mixed feature (ref: mixed, risk: not mixed), and the dependent variable is the result of FNAB (ref: benign, risk: malignant). Lesions with a single nodule on USG, where the nodule number variable contributed significantly to the model, were 3.6 times more likely to be malignant (Table 3).

**Table 3:** Malignancy estimation logistic regression analysis.

	B	*P* value	OR	95% CI for OR	
				Lower	Upper
Nodule number	1294	.025	3648	1172	11,352
Cystic structure	−2588	.334	0.075	0,000	14,297
Mix	1089	.686	2971	0.015	579,894
Constant	−4356	<.001	0.013		

## 4. Discussion

Parallel to improvements in USG, fine-needle aspiration biopsy for thyroid nodules has grown in popularity. Biopsy (FNAB) is a simple, effective and safe method, and is considered the gold standard today due to its power in determining the nature of the nodule by pathological examination. Numerous tests have been developed to reduce hesitation in uncertain cytology results. However, those tests cannot be applied in many centers because of their uncertain diagnosis and high cost.^[7]^ In this study, parameters that may be important for cytology results in thyroid USG were evaluated. Of the 532 included evaluations, 2% were found to be suspicious and malignant in cytology. Again, in our study, it was found that the number of nodules, cystic structure status, mixed features were more in favor of malignancy for findings that could be predictive of malignancy on USG, and the probability of malignancy, especially in lesions with a single nodule on USG, was found 3.6 times.

In the study of Kişioğlu et al^[8]^ 255nodules were totally evaluated in 211patients in the research performed by and the prevalence of malignancy was found to be 100% in nodules with hypoechogenic city + microcalcification + border irregularity. The probability of malignancy was found to be significantly higher in the dual combination of these 3 adverse conditions, and margin irregularity was observed to be the most predictive parameter for malignancy (odds ratio: 5.249, *P* < .001).

Deandrea et al^[9]^ In the study performed by thyroid FNAB, 46 (11.0%) of 420 cytological samples were found positive for thyroid cancer, 313 (74.5%) were negative, and 61 (14.5%) were nondiagnostic. Twenty-seven cytologically positive nodules had histologically shown malignant growth. Twelve (or 45%) of these histologically malignant lesions are not palpable, 9 (33%) are single palpable nodules, and 6 (22%) are nodules with suspicious ultrasound pattern in multinodular goiter.

In the study performed by Singaporewalla et al^[10]^, 74 of 100 cases were benign or possibly benign, 20 were suspicious for malignancy and 6 were uncertain in USG findings. The general agreement rate of FNAB and USG was reported as 83%, sensitivity as 70.6% and specificity as 90.4%, respectively.

In the study of Mandel et al^[11]^, 5 features suggesting malignant potential were reported in ultrasound features of thyroid nodules; hypoechogenic city, microcalcification, irregular or micro lobular margin, absent or irregular thick halo, and increased intramodular vascularity.

Gul et al^[12]^ In his study, 3404 nodules were examined retrospectively. The cytological results of the patients were found to be 1718 (82.5%) benign, 196 (9.4%) suspicious, 68 (3.3%) nondiagnostic and 100 (4.8%) malignant. Considering the USG features of the nodules, border irregularity, hypoechoic pattern and microcalcification were determined as risk factors predicting malignancy (Odds ratios: 63.2, 13.3, 7.03), respectively. Histopathological examination revealed a significantly higher malignancy rate in patients with solitary nodules than in patients with multiple nodules (11.7% vs 6.5%, respectively; *P* < .001). In conclusion, ultrasonographical hypoechoic pattern, microcalcification and marginal irregularity of thyroid nodules were found to be important features in determining the risk of malignancy. In our study, lesions with a single nodule on USG were found to be 3.6 times more likely to be malignant. These results were similar to those of Deandrea et al and Gul et al It is similar to the results of solitary thyroid nodules seen in studies with a higher malignant potential.

De et al^[13]^ compared sonographic features and fine needle aspiration cytology with histopathology in the diagnosis of solitary thyroid nodule. The sensitivity, specificity, accuracy, positive predictive and negative predictive value of FNAB were 80%, 90%, 85%, 86%, and 86.6%, respectively, and 80%, 47.2%, 61%, 51.3% for USG. and 77.3%. Among the individual USG parameters, microcalcification was found to be the most sensitive (80%) and specific (86%). The sensitivity of the irregular margin and longer-than-wide shape was found to be 89% and 92%, respectively.

Arpana et al^[14]^ USG features significantly associated with malignancy for thyroid lesions in his study; It has been reported that the size of the thyroid nodule is >30 mm, its borders are unclear, solid echotexture, hypoechoic lesion, microcalcification, and increased vascularity. Mohebbi et al^[15]^ compared ultrasound features with fine needle aspiration cytology in the detection of thyroid nodules in clinical practice. Sonographic features such as microcalcification, hyper echogenicity, wider than elongated shape, no halo were found to be significantly associated with malignancy. The total accuracies of calcification, echogenicity, shape, and halon were found to be 0.96, 0.92, 0.97, and 0.82, respectively. It has been stated that USG features may be a good criterion for recommending follow-up thyroid sonography and FNA cytology. Fifty-two observational studies (12,786 nodules) were included in 1 meta-analysis study. In unselected nodules, all USG features were significantly associated with malignancy at an odds ratio ranging from 1.78 to 35.7-fold, and microcalcifications, irregular edges, and a longer-than-wide shape were reported with high specificity. Lack of elasticity was found to be the only feature with the best diagnostic performance.^[16]^ In our study, a single nodule, non-mixed character, and solid character, which are among the features mentioned in the literature, seem to be related to malignancy, similarly. The reason why no significant relationship could be found in terms of malignancy with other nodule features may be due to the difference between the radiologists performing USG. The evaluation of the radiologist experienced in thyroid USG is important in guiding the FNAB procedure.

## 5. Conclusion

The main reason for the widespread use of FNAB in thyroid nodules today is the increase in sensitivity and specificity rates and the low false positive/false negative results. Unnecessary surgery can be prevented by distinguishing between FNAB and benign/malignant in thyroid nodules, and the surgical technique to be chosen is decided according to the biopsy result. It is appropriate to follow-up the nodules that are evaluated as benign with USG and FNAB at certain periods. With the information we found in our study, the high rate of malignancy, especially of single and solid nodules, should be kept in mind.

## Author contributions

Conceptualization: Lezzan Keskin.

Data curation: Lezzan Keskin, Doğu Karahan.

Formal analysis: Lezzan Keskin, Bülent Yaprak.

Funding acquisition: Doğu Karahan, Bülent Yaprak.

Investigation: Lezzan Keskin, Doğu Karahan.

Methodology: Lezzan Keskin, Bülent Yaprak.

Project administration: Lezzan Keskin, Doğu Karahan.

Resources: Lezzan Keskin, Doğu Karahan.

Software: Lezzan Keskin, Bülent Yaprak.

Supervision: Bülent Yaprak.

Validation: Doğu Karahan, Bülent Yaprak.

Writing – original draft: Lezzan Keskin, Bülent Yaprak.

Writing – review & editing: Lezzan Keskin, Doğu Karahan.
